# Seamless triple-mode luminescence thermometry in Lu_3_(Al_4_Ga_1_)O_12_:Pr: high sensitivity from 11 to 700 K

**DOI:** 10.3389/fchem.2026.1924671

**Published:** 2026-09-28

**Authors:** Justyna Zeler, Joanna Trojan-Piegza, Eugeniusz Zych

**Affiliations:** Luminescent Materials Group, Faculty of Chemistry, University of Wrocław, Wrocław, Poland

**Keywords:** LuAGG:Pr, luminescence thermometry, multimodal thermometry, Pr3+ spectroscopy, wide range luminescence thermometry

## Abstract

This study reports on the high-performance luminescence thermometry of a Lu_3_(Al_4_Ga_1_)O_12_:Pr (LuAGG:Pr) phosphor, demonstrating a remarkable operational range spanning from deep-cryogenic to high-temperature regimes (11–700 K). Its relative thermal sensitivity, *S*_r_, remains between 0.7% and 6.5% K^−1^, exceeding 1% K^−1^ over the vast majority of this interval. To overcome the sensitivity bottlenecks inherent to single-parameter thermometers, we propose a multi-mode thermometric strategy that leverages three distinct physical mechanisms. At cryogenic temperatures (11–120 K), the population redistribution among the Stark levels of the ^1^D_2_ state yields a maximum *S*_r_ of 6.5% K^−1^. In the intermediate range (120–320 K), sensing is governed by the Boltzmann-coupled ^3^P_1_ and ^3^P_0_ states, yielding a maximum *S*_r_ of 5.5% K^−1^ when using the two-level emission ratio. Crucially, we demonstrate that the integration of the intensity ratio between the interconfigurational 5d→4f and ^1^D_2_→^3^H_4_ emissions - primarily driven by multiphonon relaxation - significantly enhances performance at elevated temperatures. While Boltzmann-type distributions show declining sensitivity above room temperature, this multiphonon-driven 5d/4f ratio revitalizes the sensor, maintaining *S*_r_ > 1% K^−1^ up to 700 K, with *S*_r_ peak of 2.2% K^−1^ at 465 K. By strategically combining these three parameters, we achieve a continuous, high-sensitivity profile across the entire 11–700 K window, establishing LuAGG:Pr as one of the most versatile Pr^3+^ singly activated materials for advanced thermal sensing reported to date.

## Introduction

Over the past decade, luminescence thermometry has evolved from a niche optical technique into one of the most promising avenues for remote temperature sensing at the micro- and nanoscale. The field has witnessed remarkable progress, including the discovery of novel luminescent materials with remarkably high relative thermal sensitivities, surprisingly wide operational ranges from cryogenic to ultra-high temperatures, the demonstration of sub-degree temperature resolution in biological systems, and the successful integration of ratiometric thermometers into functional devices (Bolek et al., 2021; 2024; Brites et al., 2012; Brites et al., 2016; Kulesza et al., 2025; Macairan et al., 2019; Mi et al., 2019; Oyama et al., 2020; Piñol et al., 2019; Suo et al., 2021). These advances have positioned luminescence thermometry–a remote reading technique - as a competitive or complementary alternative to traditional contact-based methods in applications ranging from microelectronics and photonics to *in vivo* biomedical diagnostics and harsh-environment monitoring.

Despite this undeniable development, significant challenges persist in achieving robust, accurate, and versatile thermometric performance across wide temperature ranges. The most widely adopted strategy relies on thermal coupling between emitting levels, governed by the Boltzmann distribution, which provides an elegant, self-referencing ratiometric readout through the luminescence intensity ratio (LIR) with intrinsic immunity to fluctuations in excitation power, probe concentration, or optical losses. However, this approach is inherently constrained by the energy separation (Δ*E*) between the coupled electronic levels. Excessively small Δ*E* values yield low thermal sensitivity and a narrow operating range, whereas large Δ*E* values (>2000 cm^-1^) can readily lead to rapid thermal decoupling at moderate or high temperatures, resulting in a dramatic loss of sensitivity and eventual failure of the thermometric function (Labrador-Páez et al., 2018; Pessoa et al., 2025; Shen et al., 2020). Consequently, single-mechanism Boltzmann-based thermometers typically operate effectively over limited temperature windows of a few hundred kelvin, far short of the broad ranges (e.g., 1000 K or wider) demanded by existing and emerging applications and indeed offered by some phosphors but not those based on Boltzmann thermal coupling (Geitenbeek et al., 2018; Kesarwani and Rai, 2022; Kulesza et al., 2025; Wang et al., 2022; Zhong et al., 2025; Zhou et al., 2023; Zhuang et al., 2022).

To overcome these fundamental limitations and extend reliable operation across extended temperature ranges, the field increasingly recognizes the necessity of hybrid approaches that synergistically combine the Boltzmann thermal-coupling mechanism with additional temperature-dependent phenomena. Such complementary mechanisms, including phosphor band-gap adjustment, ligand-field variations, energy-transfer effects, and phase-transition-induced spectral changes, can be engineered to predominate in temperature regions where pure Boltzmann behavior weakens (Bendel and Suta, 2022; Netzsch et al., 2022; Suta et al., 2020; Yu et al., 2021). Presumably, by judiciously integrating two or more independent or semi-independent processes into a single luminescent probe, it may be possible to achieve high relative thermal sensitivity over extraordinarily wide temperature ranges while preserving the self-referencing advantages of ratiometric readout.

In this paper, we report a wide-range multichannel thermometry strategy based on Pr^3+^-doped Lu_3_(Al_4_Ga_1_)O_12_ that ensures high sensitivity from cryogenic to 700 K. This comprehensive framework integrates three distinct thermometric regimes. At deep cryogenic temperatures, up to about 120 K, the platform relies on the Boltzmann-type population distribution among the Stark levels of the ^1^D_2_ state. In the moderate temperature range, the readout shifts to thermal coupling between the ^3^P_0_ and ^3^P_1_ levels. Finally, above 300 K, the strategy exploits the luminescence intensity ratio (LIR) between the 5d→4f/^1^D_2_→^3^H_4_ transitions, which is governed by the onset of luminescence temperature quenching and multiphonon relaxation.

We have chosen the Lu_3_(Al_4_Ga_1_)O_12_:0.1mol%Pr phosphor for this study, as it demonstrates the highest resistance to thermal quenching among the Lu_3_Al_5-x_Ga_x_O_12_:0.1mol%Pr (x = 0–5) series (Trojan-Piegza et al., 2026). In addition to its chemical and thermodynamic stability, the material offers high repeatability under both UV (∼280 nm) and blue (∼450 nm) excitation. These characteristics are highly advantageous for designing a reliable luminescence thermometer that features a wide operating temperature range and high relative sensitivity.

## Materials and measurements

### Synthesis

Lu_3_(Al_4_Ga_1_)O_12_:0.1mol%Pr powder was synthesized at 1300 °C using a Li_2_SO_4_-flux-aided method described in (Bolek et al., 2021; Trojan-Piegza et al., 2026). Stoichiometric amounts of Lu_2_O_3_ and Pr_6_O_12_ (both from Stanford Materials, 5N), Al_2_O_3_ (Roth, 4N5), and Ga_2_O_3_ (abcr GmbH, 5N), were ground in an agate mortar with acetone as a wetting medium and were mixed with Li_2_SO_4_ flux (Alfa Aesar, 99.7%) (Kimura, 2011; Zeler et al., 2014). The mixture was heated for 5 h in a corundum crucible, covered with a lid. After cooling to room temperature, the flux was washed with warm distilled water, and the phosphor powder was finally dried at 80 °C in a vacuum drier for 10 h.

### Structural and morphological characterization

Phase purity of the product was verified by X-ray powder diffraction (XRD) using a Bruker D8 Advance diffractometer with CuK_α1_ radiation (λ = 1.54056 Å) and a VANTEC detector. Morphology of the powder was examined by scanning electron microscopy (SEM) using a Hitachi S-3400N electron microscope, and elemental analysis was performed using its EDAX analyzer for energy-dispersive X-ray spectroscopy (EDS).

### Photoluminescence

For luminescence experiments in the range of 11–700 K, the samples were mounted on the copper holder of a closed-cycle helium cryostat from Lake Shore Cryotronics, Inc. The spectra were taken every 25 K. For the luminescence from the ^1^D_2_ level, the step was 5 K from 5 to 200 K. Photoluminescence excitation (PLE) and emission (PL) spectra were recorded on an FLS 1000 Spectrometer from Edinburgh Instruments Ltd., equipped with a 450 W Xenon arc lamp as a continuous excitation light source and double-grating Czerny-Turner monochromators (focal length: 2 × 325 mm) in the excitation and emission line. The light was recorded with a thermoelectrically cooled (−20 °C) PMT-980 photomultiplier operating in the 185–900 nm range. Excitation spectra were corrected for incident light intensity, whereas emission spectra were additionally corrected for the grating efficiency and the photomultiplier’s spectral sensitivity. Power-dependent photoluminescence spectra were recorded by using a diaphragm built into the spectrometer to attenuate the excitation light incident on the sample.

### Characterization of thermometric performance

Temperature sensing was performed using the luminescence intensity ratio (LIR) method, grounded in the Boltzmann distribution and the integrated areas of the respective emission bands. All raw emission spectra, originally recorded in the wavelength domain, were converted to the energy scale to ensure physical consistency in the thermometric analysis. This conversion followed the standard Jacobian transformation (Mooney and Kambhampati, 2013; Mooney and Kambhampati, 2014) to preserve the spectral power distribution and the integrity of the integrated areas, which are fundamental for calculating the thermometric parameter *Δ* (Brites et al., 2012). Additionally, baseline subtraction was performed in MATLAB 2025. The thermometric parameter, *Δ*, was defined as the LIR of the integrated emission areas (Equation 1):

$$\Delta =\frac{I_{2}}{I_{1}}=A\;\exp\left(\frac{-\Delta E}{k_{B}T}\right)$$ (1)

where *I*_2_ and *I*_1_ are integrated intensities of the higher- (^3^P_1_ → ^3^H_5_) and lower-energy (^3^P_0_ → ^3^H_5_) emissions of Pr^3+^ ions, respectively. The sensing performance was evaluated based on relative thermal sensitivity, *S*_r_, and temperature resolution (δT ). For a Boltzmann-type primary thermometer, where the population of emitting levels follows a Boltzmann distribution, *S*_r_, is expressed as (Maturi et al., 2023) (Equation 2):

$$S_{r}=\frac{1}{\Delta }\;\left|\frac{\partial \Delta }{\partial T}\right|=\frac{\Delta E}{k_{B}T^{2}}$$ (2)

Under such conditions, *S*_r_ is governed by the energy gap Δ *E* and the absolute temperature *T*. Given that Δ *E* remains essentially constant across the investigated temperature range, *S*_r_ naturally increases as temperature decreases. For comparative purposes with existing literature, *S*_r_, values are reported herein at room temperature (*T*_0_). The reliability of the temperature sensor was further assessed through the temperature uncertainty ( δ *T*), which represents the minimum resolvable temperature change. This parameter is derived from the relative uncertainty of the thermometric parameter ( δΔ/Δ ) and *S*_r_ (Brites et al., 2016; Brites et al., 2019) (Equation 3):

$$\delta T=\frac{1}{S_{r}}\frac{\delta \Delta }{\Delta }$$ (3)

The relative uncertainty, δΔ/Δ , was calculated using the uncertainties of the integrated intensities ( $\delta I_{1\;}$ and $\delta I_{2\;}$ ), estimated from the ratio of baseline fluctuations to each peak intensity (Brites et al., 2016). For the sample studied, the values of δΔ/Δ were determined to be between 0.2% and 1.0%, depending on the temperature.

The evaluation of secondary thermometry performance was conducted using a rigorous methodology for calculating thermometric parameters and estimating measurement uncertainties, strictly adhering to the established protocol described in detail in the literature (Bolek et al., 2021; Bolek et al., 2024; Brites et al., 2016; Trojan-Piegza et al., 2026), which we summarize in the Supplementary Material (*Secondary thermometry methodology* section). In this framework, the relative thermal sensitivity and temperature resolution were determined by accounting for potential sources of experimental errors, including fluctuations in the detection system’s signal-to-noise ratio. Statistical propagation of uncertainty was applied to the integrated emission intensities to ensure the reliability of the calculated thermometric parameter *Δ*. This standardized approach enables a direct and meaningful comparison of our results with those of other secondary luminescent thermometers reported in the literature.

## Results and discussion

### Structure and morphology

XRD patterns and representative SEM images of Lu_3_(Al_4_Ga_1_)O_12_:0.1mol%Pr powder are shown in Supplementary Figure S1. Comparison with the Lu_3_Al_5_O_12_ reference (Trojan-Piegza et al., 2026) confirms a phase-pure cubic garnet structure. Slight shift of the diffraction peaks to lower angles reflects the increase of the interatomic distances as Ga^3+^ is larger than Al^3+^ (Shannon, 1976). The SEM images reveal non-agglomerated, fairly uniform grains with diameters ranging from 0.6 to 1.5 µm (see Supplementary Figure S1). This high crystallinity minimizes extrinsic defects and structural perturbations, ensuring that the observed luminescence properties are intrinsic to the phosphor’s composition and structure. Thus, high-quality morphology is important to the reliability of the luminescence data. Energy dispersive X-ray spectroscopy (EDS) analysis revealed a slight Ga deficiency in the fabricated Lu_3_(Al_4_Ga_1_)O_12_:Pr phosphor. The Ga/Lu atomic ratio was found to be 0.23, compared to the theoretical value of 0.33. In contrast, the Al/Lu ratio was 1.39, slightly exceeding the theoretical value of 1.32. Consequently, the total $\left(\text{Al}+\text{Ga}\right)/\text{Lu}$ ratio was 1.62, which is very close to the theoretical value of 1.67, with the difference falling within experimental uncertainty.

### Photoluminescence

Figure 1a presents the scheme of the Pr^3+^ luminescence ion and temperature-dependent emissions of the Lu_3_(Al_4_Ga_1_)O_12_:0.1mol%Pr phosphor from 11 to 700 K. The luminescence is composed of three main portions resulting from (i) Pr^3+^ 5d→4f interconfigurational transition generating broad, structured band in UV range, (ii) ^3^P_0_→^3^H_4_ transition of the dopant around 480–490 nm with additional components appearing around 530 nm (^3^P_1_→^3^H_5_), 560 nm (^3^P_0_→^3^H_5_), and above 600 nm (^3^P_0_→^3^H_6_). The latter partly overlaps with the emission from ^1^D_2_, as both appear in the red part of spectrum.

**FIGURE 1:**
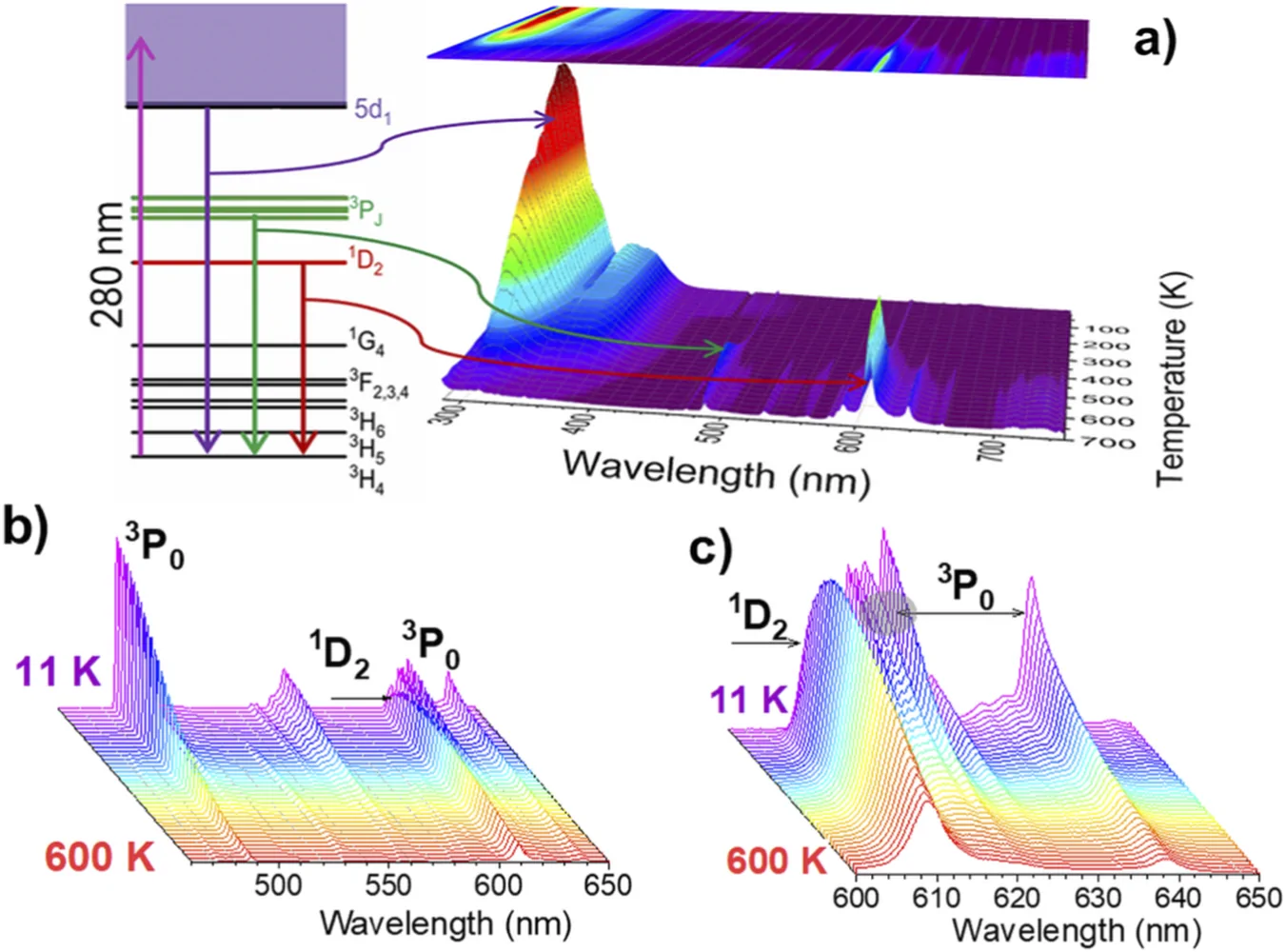
**(a)** Photoluminescence spectra of Lu_3_(Al_4_Ga_1_)O_12_:0.1mol%Pr (11–700 K) under UV excitation (280 nm, Pr^3+^ 4f → 5d), indicating the transitions responsible for UV, blue-green, and red emissions. **(b)** Corresponding temperature-dependent spectra under direct ^3^H_4_ → ^3^P_2_ excitation (450.5 nm). **(c)** Magnified view of the red spectral region highlighting the potential overlap between ^1^D_2_ and ^3^P_0_ emissions.

Figure 1b shows emission in the 11–600 K range upon ^3^H_4_→^3^P_2_ excitation at 450.5 nm. The wavelength was selected based on the high-resolution excitation spectra shown in Supplementary Figure S2. It has been shown that overlapping emissions from ^1^D_2_ and ^3^P_0_ in the red can be effectively separated using time-resolved emission spectroscopy (Sójka et al., 2020; Sójka et al., 2021; Trojan-Piegza et al., 2020). The ^3^P_0_→^3^H_J_ transitions are spin-allowed, and thus they decay by 1–2 orders of magnitude faster than ^1^D_2_→^3^H_J_. In the case of Lu_3_(Al_4_Ga_1_)O_12_:0.1mol%Pr, the ^1^D_2_ luminescence is mainly located between 604–613 nm and, in this wavelength range, does not overlap with ^3^P_0_→^3^H_4_ emission lines, which appear at yet longer wavelengths see Figure 1c (Blasse, 1989; Bolek et al., 2024). This figure clearly shows that luminescence from ^3^P_0_ weakens continuously from deep cryogenic temperatures to 600 K.

A different temperature dependence is observed for the ^1^D_2_ luminescence, see Figure 1b,c (Bolek et al., 2024; Jedoń et al., 2024; Kulesza et al., 2025; Zheng et al., 2022). Upon heating above helium temperatures, it increases in intensity up to about 200 K, and only at yet higher temperatures does it decrease continuously. Importantly, the dependence of both the ^3^P_0_ and ^1^D_2_ emissions upon 280 nm excitation is somewhat different, especially at higher temperatures (Figure 1a), due to a great role played by the interaction of the excited electron at the 5d_1_ level with the conduction band. This difference underscores the importance of excitation energy in evaluating the thermometric potential of a phosphor. Above approximately 200 K, the ^3^P_0_ emission quenches faster than the ^1^D_2_ luminescence. This aligns with the expected thermal population of the ^1^D_2_ state from ^3^P_0_ via multiphonon relaxation - a process that is negligible near helium temperatures but grows rapidly as the temperature rises (Figure 1b,c).

## Thermal population of ^1^D_2_ stark levels and its thermometric potential

The different temperature dependence of the emissions from the ^3^P_0_ and ^1^D_2_ levels prompted us to measure the ^1^D_2_ → ^3^H_4_ luminescence in the cryogenic range with higher resolution, using a smaller T-step of 5 K instead of 25 K. Figure 2a shows the scheme of the levels of interest, and the results of the measurements are presented in Figure 2b. The high-resolution spectra revealed important details not seen in the previous measurements (Figure 1c). Among others, what initially appeared to be a single luminescent line was actually a few overlapping emissions spanning 605–613 nm. The emission may be divided into two regions. The part termed “1” in Figure 2b consists, in principle, of one line, which peaks at 611.9 nm, and its intensity clearly reduces with increasing temperature. This coincides with the opposite temperature dependence of the luminescence in the range termed “2”, which gains intensity up to about 200 K. Such a dependence of the luminescence from the ^1^D_2_ level must reflect the splitting of its Stark levels in the D_2_ local symmetry of the Pr^3+^(Lu^3+^) site. Since Pr^3+^ is a non-Kramers ion, the splitting should result in five Stark levels of different energies. Obviously, the terminal ^3^H_4_ level also splits into multiple Stark sublevels. Altogether, high-resolution ^1^D_2_ → ^3^H_4_ emissions are expected to be composed of strongly overlapping components. Note that the similarity of ionic radii, identical charges, and similar chemistry make the Lu^3+^ D_2_ site in Lu_3_(Al_4_Ga_1_)O_12_:0.1mol%Pr host the only practically available location of Pr^3+^ ion. We are not aware of any literature questioning this. Due to the relatively low synthesis temperature (1300 °C), even the generation of antisite defects (Li et al., 2025; Shiran, 2016; Stanek et al., 2013; Zorenko et al., 2004; Zorenko et al., 2008; Zorenko et al., 2010) must be inefficient, as their formation requires significantly higher temperatures, close to the melting point of LuAG, which is about 2000 °C.

**FIGURE 2:**
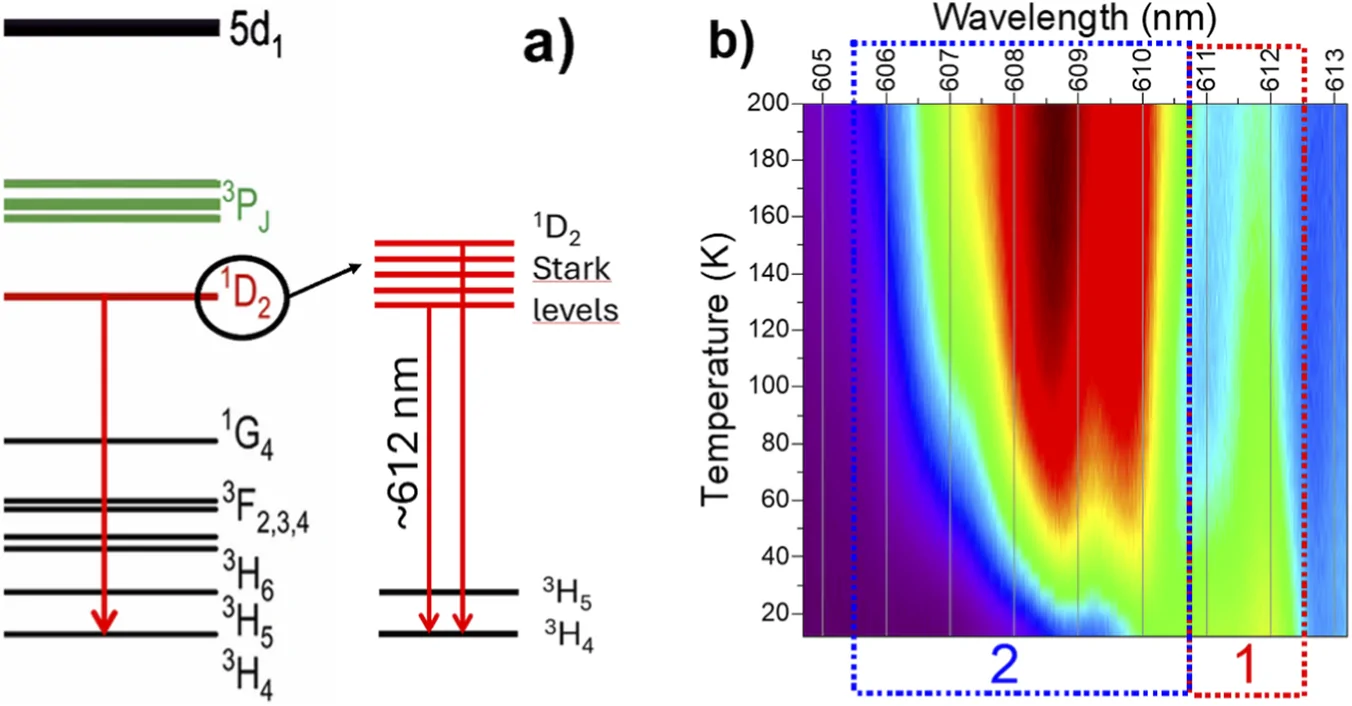
**(a)** Schematic of the ^1^D_2_ → ^3^H_4_ luminescence transitions of Pr^3+^. **(b)** Temperature evolution of the normalized photoluminescence (PL) spectra (11–200 K) under ^3^H_4_ → ^3^P_2_ excitation at 450.5 nm. Further details on intensity variations are provided in Figure 3a.

To exploit the opposing temperature dependencies of the ^1^D_2_ → ^3^H_4_ emission, parts “1” and “2” of the spectra were integrated separately (Figure 2). Figure 3 summarizes these data and presents their analyses regarding thermometric performance. Intensities of parts “1” and “2” and resulting ratio across the 11–200 K range are shown in Figures 3a,b, respectively. The inset in Figure 3b presents the ln*Δ*(1/*T*) dependence, and the non-linear course of the dependence indicates that intensities “1” and “2” do not follow the Boltzmann distribution. However, the slightly narrower temperature range of 50–200 K presented in Figure 3c does meet the Boltzmann distribution very well, with Δ*E* = 48.1 ± 0.7 cm^-1^. The potential of the Lu_3_(Al_4_Ga_1_)O_12_:0.1mol%Pr luminescence thermometer is highlighted in Figure 3d by a relative thermal sensitivity (*S*_r_) reaching 6.7% K^−1^ at 11 K. Notably, *S*_r_ remains above 1% K^−1^ up to 80 K, only falling below 0.5% K^−1^ after 130 K. This performance profile, coupled with a temperature uncertainty below 1 K (Figure 3d), positions this phosphor as a highly competitive candidate for high-resolution deep cryogenic temperature sensing (Bolek et al., 2024; Jedoń et al., 2024; Kulesza et al., 2025; Zheng et al., 2022). Table 1 lists such types of reported luminescence thermometric phosphors and their basic properties in the deep cryogenic range.

**FIGURE 3:**
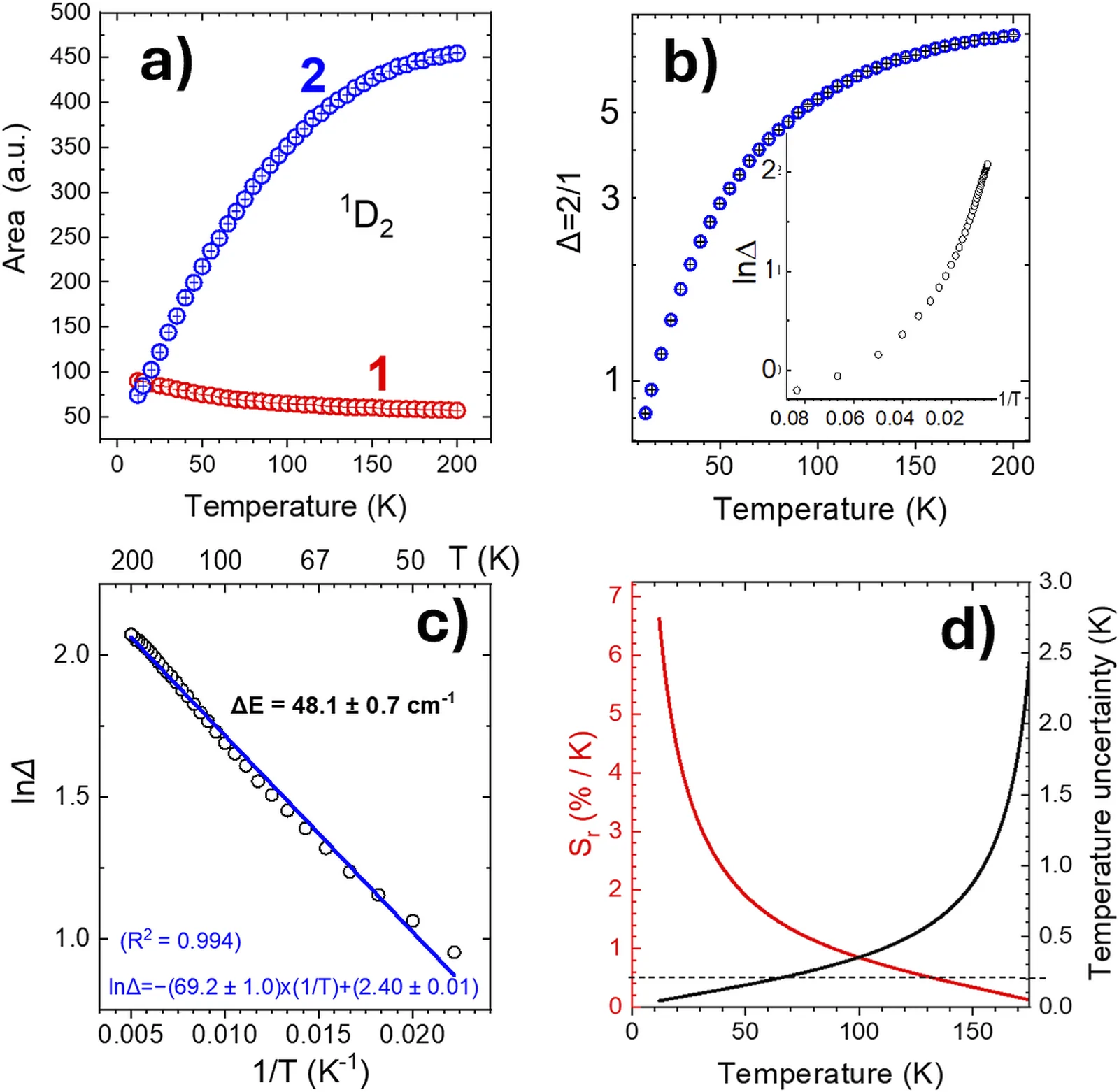
**(a)** Temperature dependence of the emission intensities for the ^1^D_2_ → ^3^H_4_ transition, integrated over the low-energy (1) and high-energy (2) Stark components (see Figure 2b). **(b)** Luminescence intensity ratio (Δ) of the (2) and (1) areas of the ^1^D_2_ → ^3^H_4_ emission, **(c)** The linear part of Δ from **(b)** covering the range of 50–200 K, **(d)** Relative thermal sensitivity (S_r_) and corresponding temperature uncertainty derived from the intensity ratio of the (1) and (2) components. Refer to Figure 2 for spectral definitions.

**TABLE 1:** Maximum relative thermal sensitivity (S_m_) and the temperature at which it occurs (T_m_) reported for Pr-doped phosphors operating in the cryogenic range (≤100 K).

Material	*S*_m_ (%·K^−1^)	*T*_m_(K)	Ref.
Lu_3_(Al_1_Ga_4_)O_12_:Pr^3+^	6.5	11	This work
Lu_3_Al_5_O_12_:Pr^3+^ (LuAG:Pr)	5.26	12	Trojan-Piegza et al. (2026)
Lu_3_(Al_3_Ga_2_)O_12_:Pr^3+^	3.08	15	
Lu_3_(Al_2_Ga_3_)O_12_:Pr^3+^	2.74	77	
Lu_3_(Al_1_Ga_4_)O_12_:Pr^3+^	4.9	30	
Lu_3_Ga_5_O_12_:Pr^3+^	2.8	40	
YAG:Pr^3+^	1.7	90	Bolek et al. (2021)
Y_3_Al_4_Ga_1_O_12_:Pr^3+^	1.4	100	
Y_3_Al_3_Ga_2_O_12_:Pr^3+^	0.75	50	
Y_3_Al_2.5_Ga_2.5_O_12_:Pr^3+^	0.9	100	
Y_3_Al_2_Ga_3_O_12_:Pr^3+^	1.2	100	
Y_3_Al_1.5_Ga_3.5_O_12_:Pr^3+^	1.4	85	
Y_3_Al_1_Ga_4_O_12_:Pr^3+^	3.6	60	
Sr_2_GeO_4_:Pr^3+^	9	22	Sójka et al. (2019)
Sr_2_Ge_0.75_Si_0.25_O_4_:Pr^3+^	9.2	65	
Lu_2_Ge_0.75_Si_0.25_O_5_:Pr^3+^	3.54	17	Sójka et al. (2020)
Sr_3_Y_2_Ge_3_O_12_:Pr^3+^	1.5	100	Bolek et al. (2024)
Sr_3_Sc_2_Ge_3_O_12_:Pr^3+^	1.4	100	
BaSc_2_Ge_3_O_10_:Pr^3+^	2.3	82	Li et al. (2023)
Ca_3_Sc_2_Ge_3_O_12_:Pr^3+^	0.32	20	Kulesza et al. (2025)
Ca_3_Sc_2_Si_3_O_12_:Pr^3+^	0.1	90	
Y_2_Si_0.25_Ge_0.75_O_5_:Pr^3+^	2.25	100	Sójka et al. (2021)
La_2_MgTiO_6_:Pr^3+^	0.15	77	Shi et al. (2017)
CaTiO_3_Pr^3+^	0.2	50	Sharma, (2023)
CaNb_2_O_6_:Pr^3+^	11.3	60	Li et al. (2024)
Ca_3_(Ta,Ga)_5_O_12_:Pr^3+^	1.64	10	Gheorghe et al. (2022)
LaF_3_:Pr^3+^	3.1	80	Pudovkin et al. (2017)
NaYF_4_:Pr^3+^	25	50	Zhou et al. (2014)
LiTaO_3_:Pr^3+^	0.5	100	Long et al. (2025)
Li_2_CaTa_2_O_7_ Pr^3+^	2.2	100	Yin et al. (2025)
SrLu_2_O_4_:Pr^3+^	0.75	78	Wang et al. (2020)
SrY_2_O_4_:Pr^3+^	0.8	100	
Ba_3_Lu_4_O_9_:Pr^3+^	1.3	100	Han et al. (2023)
Ba_3_Y_4_O_9_:Pr^3+^	2	100	
Ba_4_Y_3_F_17_:Pr^3+^	11	80	Pudovkin et al. (2020)
La_2_Zr_2_O_7_:Pr^3+^	0.35	100	Jahanbazi et al. (2022)
La_0.4_Gd_1.6_Zr_2_O_7_:Pr^3+^	0.5	17–100	Trojan-Piegza et al. (2020)

## ^3^P_0_ to ^3^P_1_ thermal population and its thermometric potential

The thermal coupling of the Pr^3+^ ^3^P_J_ levels is a well-known effect and has frequently been reported to be useful for thermometric purposes (Jahanbazi et al., 2022; Runowski et al., 2019; Tang et al., 2022; Wang et al., 2022). Emissions from ^3^P_0_ are observed typically around 480–500 nm (transition to ^3^H_4_), near 560 nm (^3^H_5_), and above about 610 nm (^3^H_6_). This process is schemed in Figure 4a. In Lu_3_(Al_4_Ga_1_)O_12_:0.1mol%Pr, the most intense is the ^3^P_0_ → ^3^H_4_ luminescence, as seen in Figure 4b. However, the most favorable conditions for Boltzmann-type thermometric analysis were observed for luminescence in the 520–580 nm range (^3^P_0,1_ → ^3^H_5_), as shown in Figure 4c. While less intense, the ^3^P_0_ → ^3^H_5_ emission was accompanied by a well-exposed, relatively strong, and well-separated component from ^3^P_1_ at about 532 nm. Typically, the two levels are separated by about 500–600 cm^-1^, which allows effective thermal coupling above about 100–150 K.

**FIGURE 4:**
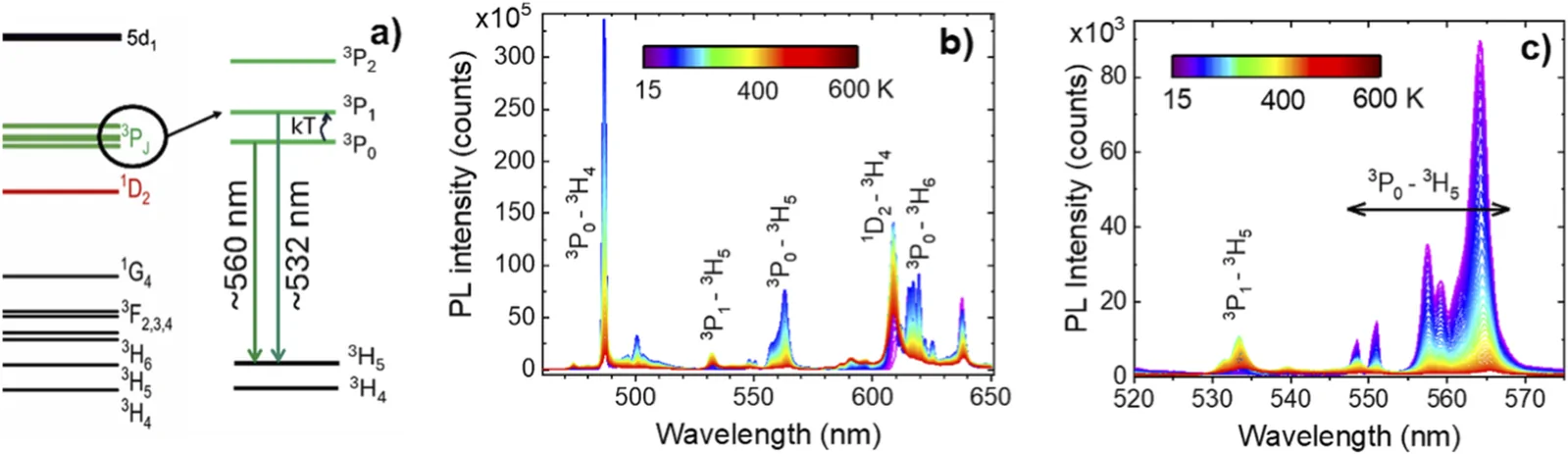
**(a)** Energy level diagram of Pr^3+^ detailing the ^3^P_J_ (J = 0, 1, 2) multiplets. **(b)** Temperature-dependent emission spectra (11–600 K) under ^3^H_4_ → ^3^P_2_ excitation at 450.5 nm. **(c)** Magnified view of the green luminescence region, highlighting the transitions utilized for ratiometric thermometry.

The emission spectra at 11 K and 300 K (Figure 5a) confirm that the band at 532 nm originates from the thermally induced population of the ^3^P_1_ level, which contributes significantly to the luminescence at room temperature. Based on these spectra, the energy gap between the thermally coupled ^3^P_0_ and ^3^P_1_ states was determined to be Δ*E* = 516.2 cm^-1^. This value serves as a fixed parameter for fitting the ^3^P_1_/^3^P_0_ intensity ratio using the Boltzmann distribution (see Equation 1).

**FIGURE 5:**
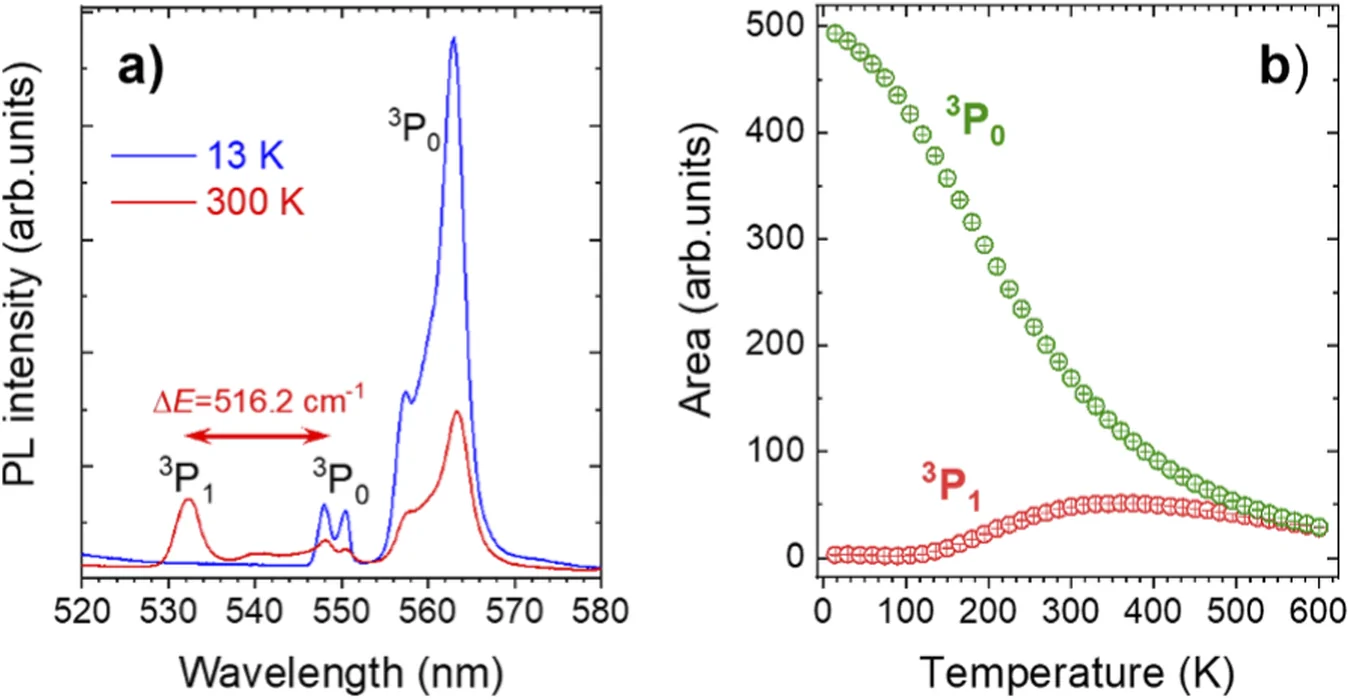
**(a)** Comparison of Pr^3+^ green luminescence at 11 K and 300 K, highlighting the emergence of ^3^P_1_ emission at higher temperatures due to thermally induced population from the ^3^P_0_ level. **(b)** Thermal evolution of the integrated luminescence intensities for the ^3^P_0_ and ^3^P_1_ levels.

The thermal evolution of the individual ^3^P_1_ and ^3^P_0_ emissions is shown in Figure 5b. While ^3^P_1_ luminescence becomes detectable only above 100 K, its intensity increases consistently with temperature at the expense of the ^3^P_0_ emission. This inverse relationship follows the Boltzmann distribution law governing thermally coupled levels. However, the decline in ^3^P_0_ intensity is more pronounced than the growth of the ^3^P_1_ signal. This is attributed to the simultaneous thermally stimulated feeding of the ^1^D_2_ state via multiphonon relaxation from ^3^P_0_ (Figure 1; de Mello Donega et al., 1995; Dorenbos, 2023; Förster et al., 2025).

Consequently, the experimental data were fitted using the Boltzmann-type sigmoidal model (Equation 1), normalized to the reference temperature proposed by (Balabhadra et al., 2017) as described by (Equation 4).

$$\frac{1}{T}=\frac{1}{T_{0}}-\frac{k_{B}}{\Delta E}\ln\left(\frac{\Delta }{\Delta_{0}}\right)$$ (4)

where *k*_B_ denotes the Boltzmann constant, Δ*E* is the energy separation between the involved levels (^3^P_0_ and ^3^P_1_), and *Δ* = *I(* ^3^P_1_)/*I(* ^3^P_0_) represents the thermometric parameter, with *Δ*_*0*_ defined as the intensity ratio at the reference temperature *T*_*0*_ (300 K). The relation between temperature and the ^3^P_1_/^3^P_0_ intensity ratio is given in Figure 6a together with the fit of the experimental data using Equation 4 (red line). To evaluate the applicability of the Boltzmann distribution between the ^3^P_1_ and ^3^P_0_ levels ln*Δ* vs. 1/*T* was plotted and is presented in Figure 6b. In the 110–600 K temperature range, the ^3^P_1_/^3^P_0_ ratio follows the Boltzmann distribution very well with Δ*E* = 477.7 ± 6.5 cm^-1^, while at lower temperatures experimental data diverge from the fit. Note that this is the temperature range where the thermal population of the ^3^P_1_ level must be very limited, and the emission intensity from this level is greatly reduced. This behavior is commonly observed in Boltzmann-type thermometers at the low-temperature limit of their operating range, when the higher-energy level is nearly unpopulated. It does not indicate a breakdown of the underlying thermal coupling mechanism, but it rather reflects the practical sensitivity limits of the technique at very low temperatures.

**FIGURE 6:**
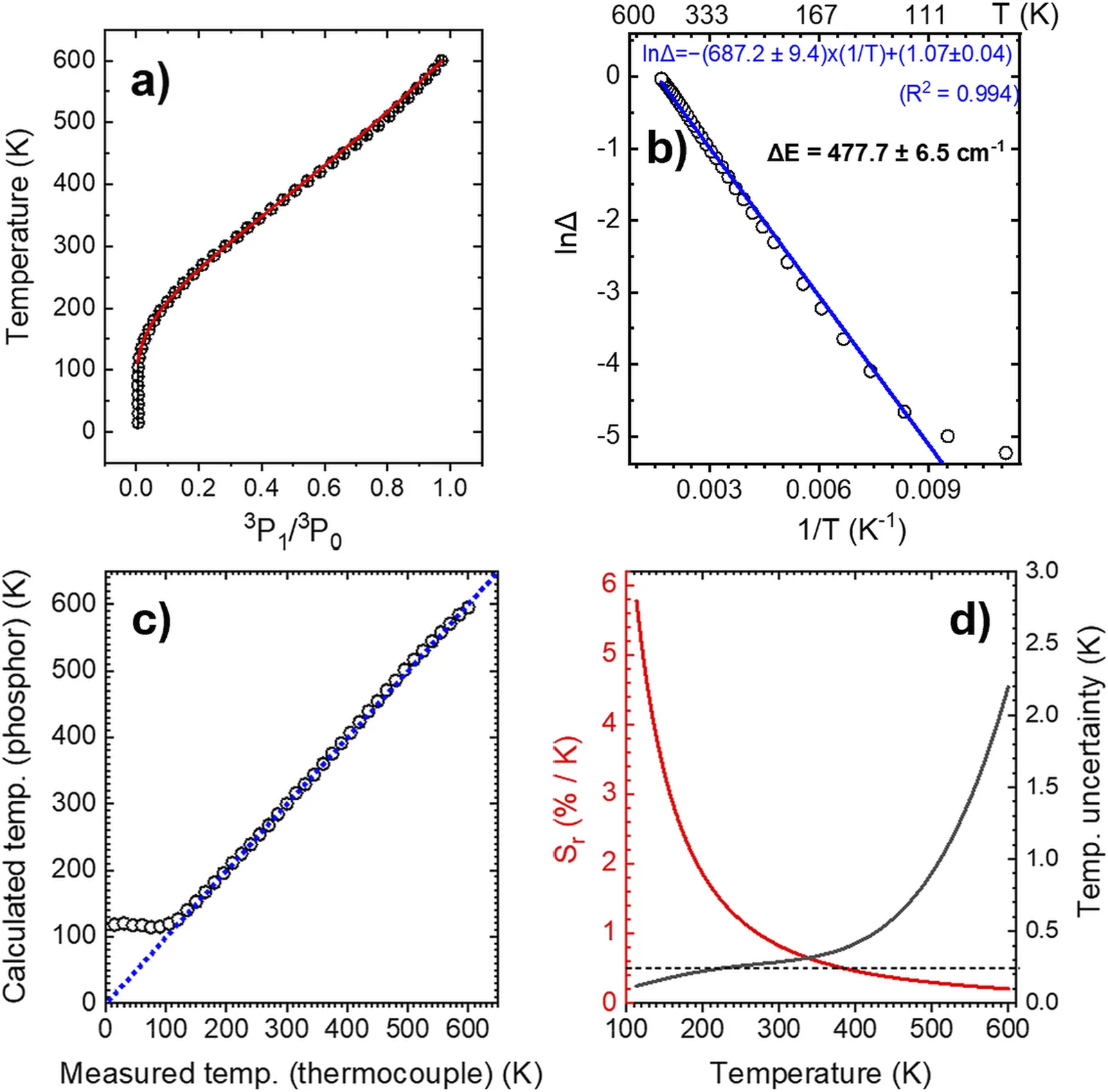
**(a)** Dependence of the ^3^P_1_/^3^P_0_ intensity ratio on temperature and the fit of the experimental data with Equation 4 (red line). **(b)** Dependence of lnΔ vs. 1/T in the ∼100–600 K range. Linearity in the 110–600 K range proves the Boltzmann distribution. **(c)** Comparison between the temperatures calculated from the luminescence spectra of the phosphor and the reference temperatures measured by the thermocouple. The dashed blue line represents the ideal y = x correlation. A high degree of linearity is observed across the 125–600 K range, demonstrating the accuracy and reliability of the proposed thermometric model in Lu_3_(Al_4_Ga_1_)O_12_:0.1mol%Pr. **(d)** Dependence of the relative thermal sensitivity (black line) and temperature uncertainty (red) with temperature.

Next, to evaluate the reliability of the calibration model, the experimental *Δ* values were converted back into temperature readings using the derived relationship. The high correlation between these calculated values and the set temperatures is demonstrated in Figure 6c, confirming the robustness of the calibration curve. Note that exciting the phosphor with radiation from the Xe lamp does not cause any measurable local rise in sample temperature, as the excitation density is relatively low. This situation could change if focused laser irradiation were used, as Balabhadra et al. (2017) showed in their experiments. To confirm this difference, we recorded emission spectra while increasing the excitation power by a factor of 6 (using the IRIS functionality in the spectrometer software). As shown in Supplementary Figure S3, no changes in relative intensities were observed, confirming that these relatively low excitation powers do not introduce artifacts related to sample heating. Long-term irradiation stability under 280 nm radiation was also assessed during a 70 h continuous experiment. The results are presented in Supplementary Figure S4. While an ∼8% decrease in the overall luminescence intensity was observed, the relative intensities of the various bands remained nearly constant.

Figure 6d presents a comprehensive metrological characterization of the Lu_3_(Al_4_Ga_1_)O_12_:0.1mol%Pr luminescence thermometric phosphor over a wide operating range from 125 to 600 K using the ^3^P_1_/^3^P_0_ intensity ratio. The black curve shows the temperature dependence of the *S*_r_, which reaches a maximum value exceeding 5.5% K^−1^ in the cryogenic regime. This high thermal sensitivity at low temperatures gradually scales down, which is characteristic of Boltzmann-type thermometers, to approximately 0.2% K^−1^ at 600 K. Complementary to this, the temperature uncertainty, δT, represented by the red line, does not exceed 0.5 K up to approximately 400 K, before showing a moderate increase towards 2.2 K at the upper thermal limit.

Using the Boltzmann distribution and the calculated energy gap Δ*E* = 516.2 cm^-1^, the theoretical optimal performance window - defined as the interval of maximum thermodynamic response - was found to span 217.6–371.4 K. This range is ideal for near-ambient physiological and environmental monitoring (Suta and Meijerink, 2020). However, experimental data (Figure 6c) show that the Lu_3_(Al_4_Ga_1_)O_12_:0.1mol%Pr thermometer remains highly functional well beyond these limits. Specifically, in the low-temperature regime down to 110 K, the sensor exhibits increasing sensitivity and improved measurement precision (Figure 6d). Furthermore, the correlation between the emission-derived temperature and the reference thermocouple remains remarkably linear over the range from 125 K to 600 K (Figure 6c). Consequently, while the theoretical optimum is narrower, the practical operating range for reliable, high-accuracy sensing using the ^3^P_1_/^3^P_0_ ratio extends from 125 K to 400 K.

## Temperature-dependent branching of 5d→4f and 4f→4f emissions and its thermometric potential

The above-discussed changes in intensities of the various 4f→4f luminescent features were driven by the Boltzmann distribution of excited electrons between thermally coupled levels. Figure 1, however, showed that while the 5d→4f luminescence intensity decreases with increasing temperature, emissions from the ^3^P_0_ and ^1^D_2_ levels tend to increase, recovering part of the energy nonradiatively lost from the 5d. Such changes are highly attractive for thermometric purposes, and below we qualitatively analyze the feasibility of using the 5d→4f/4f→4f intensity ratio as a thermometric parameter, Δ. Using the ^3^P_0_→^3^H_4_ intensity instead of ^1^D_2_→^3^H_4_ appeared less favorable and is not discussed.

The integrated intensity of the broadband 5d→4f luminescence is quite stable up to about 170 K, then weakens with different dynamics up to 700 K, where it shows only remnant intensity, see Figure 7a. On the contrary, the luminescence from the ^1^D_2_ level is stable but very weak up to about 300 K, then its intensity increases to approximately 600 K and remains stable to 700 K. Consequently, the 5d→4f/^1^D_2_→^3^H_4_ intensity ratio remains constant in the 11–270 K range and continuously decreases up to 700 K. The experimental data could be easily and accurately fitted using Supplementary Equation S3 Relative thermal sensitivity, *S*_*r*_, could then be reliably calculated in the 275–700 K temperature range (Figure 7b), presenting a value of 0.7% K^−1^ at 270 K and growing to a maximum of 2.3% K^−1^ at 460–470 K. Above 470 K, the sensitivity slowly decreases, reaching 1.25% K^−1^ at 700 K. Thus, using the 5d→4f/^1^D_2_→^3^H_4_ intensity ratio extends the operating range of Lu_3_(Al_4_Ga_1_)O_12_:0.1mol%Pr to 700 K.

**FIGURE 7:**
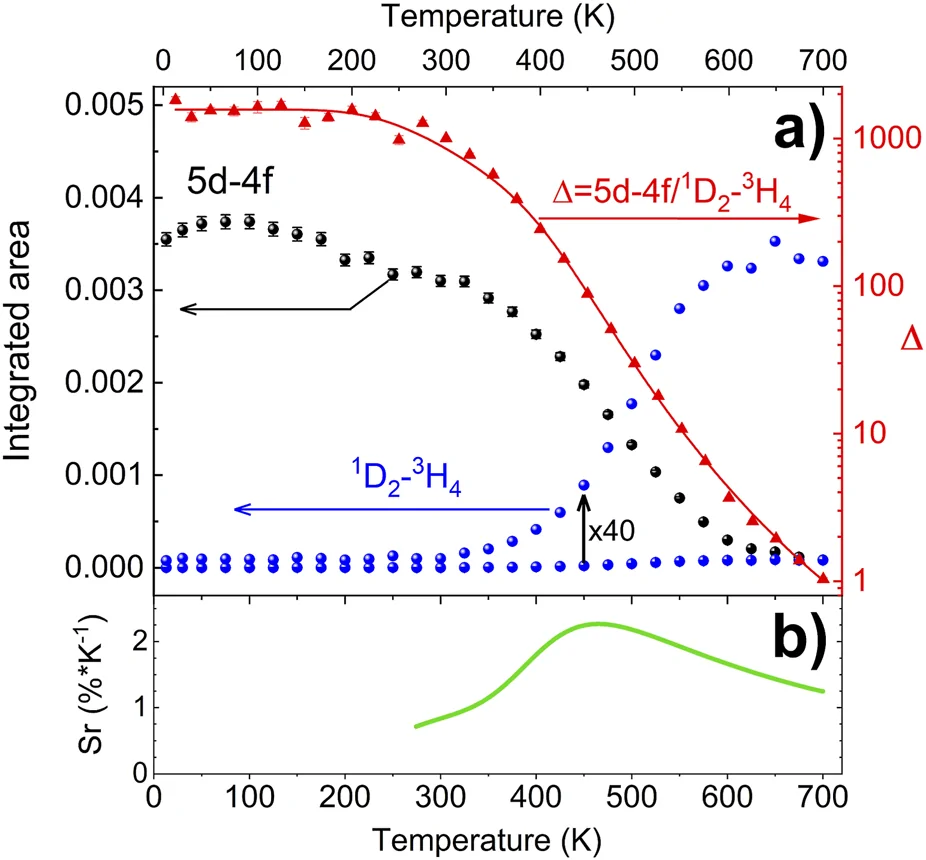
**(a)** Integrated intensities of the 5d→4f (black points) and ^1^D_2_→^3^H_4_ (blue) emissions and the ratio of the intensities (red). **(b)** The relative thermal sensitivity using the 5d→4f/^1^D_2_→^3^H_4_ intensity ratio.

## Summary and conclusions

In this study, we have demonstrated a comprehensive thermometric analysis of Lu_3_(Al_4_Ga_1_)O_12_:0.1mol%Pr, integrating classical Boltzmann-type population distributions with the distinct dynamics of interconfigurational transitions. By strategically leveraging three independent thermometric parameters, we have overcome the intrinsic sensitivity limitations that typically plague single-mode thermometers.

### Multi-mode sensitivity analysis

As illustrated in Figure 8, the relative thermal sensitivity (*S*_r_) profile is characterized by a seamless functional relay of three distinct mechanisms.

- Boltzmann-induced Stark Level Redistribution (red line): The intensity ratio between the Stark components of the ^1^D_2_→^3^H_4_ transition provides remarkable precision in the deep-cryogenic regime, peaking at 6.5% K^−1^ at 11 K.
- Boltzmann-Coupled States (green line): As the Stark sensitivity diminishes above 120 K, the ratio between the thermally coupled ^3^P_1_ and ^3^P_0_ levels becomes predominant. This mechanism maintains a robust *S*_r_ of 5.8% K^−1^ at 120 K and sustains reliable sensing resolution up to room temperature, reaching 0.7% K^−1^.
- Hybrid Interconfigurational Dynamics (violet line): Crucially, above 300 K, where traditional Boltzmann-based sensitivities reach their floor, the 5d→^3^H_J_/^1^D_2_→^3^H_4_ intensity ratio takes over. Driven by multiphonon relaxation, this mechanism revitalizes sensor performance, maintaining *S*_r_ > 1% K^−1^ up to 700 K and reaching a high-temperature peak of 2.2% K^−1^ at 465 K.

**FIGURE 8:**
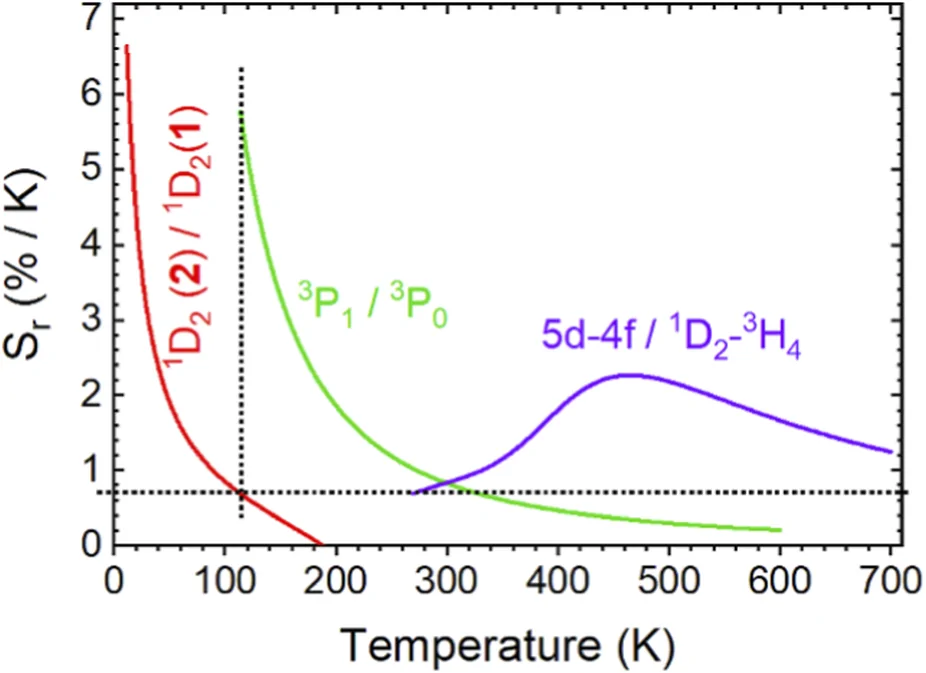
Temperature dependence of the relative thermal sensitivity S_r_ for the three distinct thermometric parameters of the Lu_3_(Al_4_Ga_1_)O_12_:0.1mol%Pr phosphor. The red curve corresponds to the intensity ratio of Stark components within the ^1^D_2_→^3^H_4_ transition, providing high sensitivity in the cryogenic regime. The green curve represents the ratiometric response of the thermally coupled ^3^P_1_ and ^3^P_0_ levels, while the violet curve depicts the thermal sensitivity derived from the 5d→4f/^1^D_2_→^3^H_4_ intensity ratio. The dotted horizontal line indicates a sensitivity threshold of 0.7% K^−1^, highlighting continuous high-performance sensing capability across the entire 11–700 K range. The vertical dotted line marks the transition point at which the ^3^P_1_/^3^P_0_ coupling compensates for the declining performance of the ^1^D_2_ Stark levels, ensuring a seamless sensing relay.

### The advantage of hybrid mechanisms

A key finding of this work is the admirable efficacy of combining Boltzmann-type thermometry with multiphonon relaxation processes. While the first two parameters rely on equilibrium population distributions, the 5d/f-f ratio is governed by a fundamentally different mechanism: the temperature-dependent partitioning of energy from the 5d_1_ level via non-radiative, multi-phonon energy dissipation.

By integrating this multiphonon-driven process, we extend the high-sensitivity regime far beyond the limits of pure Stark and 4f state Boltzmann-type coupling. This hybrid approach yields an *S*_r_ exceeding 1% K^−1^ from 320 K up to 700 K, with a meaningful high-temperature maximum of 2.2% K^−1^ at 465 K.

### Impact and outlook

In conclusion, the combined effect of these three parameters allows for reliable, high-resolution temperature sensing across an extensive range from 11 to 700 K. By maintaining a sensitivity of at least 0.7% K^−1^ (and exceeding 1% K^−1^ over nearly the entire range), this phosphor overcomes the sensitivity gap common in standard materials. Consequently, the investigated garnet emerges as a premier candidate for advanced thermal sensing, offering one of the widest and most precise operational windows reported to date for praseodymium-activated materials.

## Data Availability

The raw data supporting the conclusions of this article will be made available by the authors, without undue reservation.
