# Supplementary material for: Seamless triple-mode luminescence thermometry in Lu3(Al4Ga1)O12:Pr: high sensitivity from 11 to 700 K
Source: Front Chem. 2026 Sep 28;14:1924671. doi: 10.3389/fchem.2026.1924671 (PMC13655073; doi:10.3389/fchem.2026.1924671)
Supplement: Supplementary file 1 [file Supplementaryfile1.docx]

SUPPLEMENTARY INFORMATION

**FIGURE CAPTIONS**

[**Figure S1.** SEM image and X-ray diffraction pattern of Lu_3_Al_4_Ga_1_O_12_:Pr(0.1mol%) garnet at 298 K.	2](#_Toc236497477)

[**Figure S2.** High-resolution PLE spectra of Lu_3_Al_4_Ga_1_O_12_:Pr(0.1mol%) garnet recorded at room temperature (RT) (blue line) and at 11 K (reddish line).	3](#_Toc236497478)

**Figure S3.** Normalized luminescence spectra recorded upon 280 nm excitation using different
excitation powers adjusted by the IRIS function of the spectrometer management software……………..3
**Figure S4.** Comparison of luminescence spectra taken every 5 hours over 70 hours at 450 K upon 280 nm excitation………………………………………………………………………………………………………………………………………4

[**Secondary thermometry methodology.**	5](#_Toc226724230)

**Figure S1.** SEM image and X-ray diffraction pattern of Lu_3_Al_4_Ga_1_O_12_:Pr(0.1mol%) garnet at 298 K.

**Figure S2.** High-resolution PLE spectra of Lu_3_Al_4_Ga_1_O_12_:Pr(0.1mol%) garnet recorded at room temperature (RT) (blue line) and at 11 K (reddish line).

**Figure S3.** Normalized luminescence spectra recorded upon 280 nm excitation using different excitation powers adjusted by the IRIS function of the spectrometer management software.

**Figure S4.** Comparison of luminescence spectra taken every 5 hours over 70 hours at 450 K upon 280 nm excitation.

***Secondary thermometry methodology***

The calculations were performed using the procedure described by (Brites et al., 2016), (Bolek et al., 2021) and (Trojan-Piegza et al., 2026). Since the observed emission lines are of different bandwidths, the Jacobian transformation REF was applied, and the calculation of the thermometric parameter was made from the emission spectra represented as a function of energy (eV) (Mooney and Kambhampati, 2013; Brites et al., 2018; Suta and Meijerink, 2020). The background removal as the dominant source of uncertainty in our analysis was applied, and the integrated areas of the transitions were calculated according Eq. (S1):

$I_{i}=\int_{E_{1}}^{E_{2}} f\left( E,T \right)dE$.	(S1)

The wavelength ranges used to calculate the integrated intensities are presented in (Trojan-Piegza et al., 2026). Thermometric parameter Δ is defined by the ratio of integrated intensities of two particular emission bands as:

$\Delta= \frac{I_{2}}{I_{1}}$	,	(S2)

where *I_2_* denotes the higher-energy emission (5d4f$\to$^3^H_4_) and *I_1_* the lower-energy emission (^1^D_2_$\to$^3^H_4_)*.* Δ was fitted with the Mott-Seitz (Seitz, 1939) approach:

$\Delta_{i}\left( T \right)\approx\frac{\Delta_{0}}{1+\sum_{i=1}^{2} \alpha_{i} exp \left( - \frac{\Delta E_{i}}{k_{B}T} \right)}$,	(S3)

where *Δ*_0_ represents the *Δ* parameter in the *T* → 0 K limit, *α*_i_ is the ratio between the radiative and the nonradiative transition rates of the *i^th^* deactivation channel, and Δ*E_i_* is the energy barrier for the *i^th^* process.

The uncertainty in the Δ determination was calculated with eq. (S4):

${\delta\Delta}_{i}(T)=\sqrt{\left( \frac{\delta I_{1}}{I_{1}} \right)^{2}+\left( \frac{\delta I_{2}}{I_{2}} \right)^{2}}\cdot\Delta_{i}(T)$,	(S4)

where *I*_1_ and *I*_2_ are the integrated intensities of the two corresponding bands, *δI*_1_ and *δI*_2_ are errors of the integrated areas, and *δI/I* is thus calculated based on signal-to-noise values. The relative thermal sensitivity (*S*_r_) and the temperature uncertainty (*δ*T) were calculated according to:

$S_{r}=\frac{1}{\Delta} \left| \frac{\partial\Delta}{\partial T} \right|$	(S5)

$\delta T= \frac{1}{S_{r}}\frac{\delta\Delta}{\Delta}$.	(S6)

The operating range of the luminescence thermometer was defined based on the criterion that the difference between consecutive values of Δ must exceed the temperature uncertainty, δΔ. Furthermore, data points for which a single Δ value corresponds to more than one temperature were excluded from the operating range.

***References***

Bolek, P., Zeler, J., Brites, C. D. S., Trojan-Piegza, J., Carlos, L. D., and Zych, E. (2021). Ga-modified YAG:Pr^3+^ dual-mode tunable luminescence thermometers. *Chemical Engineering Journal* 421, 129764. doi: 10.1016/j.cej.2021.129764

Brites, C. D. S., Balabhadra, S., and Carlos, L. D. (2018). Lanthanide-Based Thermometers: At the Cutting-Edge of Luminescence Thermometry. *Adv. Opt. Mater.* 1801239, 1801239.
doi: 10.1002/adom.201801239

Brites, C. D. S., Millán, A., and Carlos, L. D. (2016). Lanthanides in Luminescent Thermometry. *Handbook on the Physics and Chemistry of Rare Earths* 49, 339–427. doi: 10.1016/bs.hpcre.2016.03.005

Mooney, J., and Kambhampati, P. (2013). Get the basics right: Jacobian conversion of wavelength and energy scales for quantitative analysis of emission spectra. *Journal of Physical Chemistry Letters* 4, 3316–3318. doi: 10.1021/jz401508t

Seitz, F. (1939). An interpretation of crystal luminescence. *Transactions of the Faraday Society* 35,
74–85. doi: 10.1039/tf9393500074

Suta, M., and Meijerink, A. (2020). A Theoretical Framework for Ratiometric Single Ion Luminescent Thermometers—Thermodynamic and Kinetic Guidelines for Optimized Performance. *Adv. Theory Simul.* 3, 1–32. doi: 10.1002/adts.202000176

Trojan-Piegza J., Zeler J., Zych E. (2026). Excitation- and Composition-Driven Tuning of Thermometric Response in Lu_3_(Al,Ga)_5_O_12_:Pr Garnets. *J. Mater. Chem. C Mater.* doi: 10.1039/D6TC00884D
